# The efficacy of calcitriol treatment in non-alcoholic fatty liver patients with different genotypes of vitamin D receptor FokI polymorphism

**DOI:** 10.1186/s40360-021-00485-y

**Published:** 2021-04-07

**Authors:** Hamid Yaghooti, Fatemeh Ghanavati, Seyed Saeed Seyedian, Bahman Cheraghian, Narges Mohammadtaghvaei

**Affiliations:** 1 Hyperlipidemia Research Center, Ahvaz Jundishapur University of Medical Sciences, Ahvaz, Iran; 2 Alimentary Tract Research Center, Ahvaz Jundishapur University of Medical Sciences, Ahvaz, Iran; 3 Department of Biostatistics and Epidemiology, School of Public Health, Ahvaz Jundishapur University of Medical Sciences, Ahvaz, Iran; 4 Department of Medical Laboratory Sciences, School of Allied Medical Sciences, Ahvaz Jundishapur University of Medical Sciences, Ahvaz, Iran

**Keywords:** Non-alcoholic fatty liver disease, Calcitriol, Vitamin D receptor, FokI polymorphism

## Abstract

**Background:**

Vitamin D deficiency is prevalent in patients with non-alcoholic fatty liver disease (NAFLD), but there are debates on the usefulness of vitamin D treatment. The interindividual variations in response may be due to different genetic backgrounds.

The present study evaluated the efficacy of calcitriol treatment in NAFLD patients with regard to the vitamin D receptor (VDR) genotypes of FokI polymorphism.

**Methods:**

The study was conducted on 128 NAFLD patients randomly divided into two groups and were subjected to intervention with 0.25 mcg calcitriol/day or placebo for 4 months, while anthropometric parameters, glycemic status, lipid profiles, inflammatory markers, liver enzymes, and fatty liver indices were measured. The ARMS-PCR method was used to genotype the VDR FokI polymorphism.

**Results:**

Calcitriol treatments along with weight loss and diet recommendations decreased the liver enzymes (AST, ALT, and ALP, *p* < 0.001 for all) and fatty liver indices (HSI, *p* < 0.01 and APRI, *p* < 0.001), compared to the baseline. But when the calcitriol effects were compared to the placebo group, only ALP decrease remained significant (17.5 IU. *P* = 0.02). The prevalent FokI variants in our population were FF (53.1%) and Ff genotype (45.3%). No significant interaction of FokI variants to the calcitriol effects was found except for ALP. The decrease in the ALP activity was higher in calcitriol-received patients with the Ff genotype (*p* = 0.05).

**Conclusions:**

The FF and Ff variants of VDR FokI polymorphism did not interact with the effects of calcitriol on fatty liver, but the ALP was more responsive in subjects with the Ff variant.

**IRCT registration number:**

IRCT2017053034222N1 Registration date: 2017-06-28 - Retrospectively registered, https://en.irct.ir/trial/26203

## Background

Non-alcoholic fatty liver disease (NAFLD) is a prevalent cause of liver disease in the world [1]. The disease initiates with relatively mild steatosis and may progress to non-alcoholic steatohepatitis (NASH), fibrosis, and finally hepatocellular carcinoma (HCC) [2]. Weight loss, exercise, and diet modification are proposed as primary measures for NAFLD management [3]. It is a need to find novel and alternative drug candidates for the treatment and prevention of NAFLD in high-risk populations.

Vitamin D has crucial roles in manipulating the immune responses, cell proliferation, regulation of the inflammatory processes, and metabolic diseases [4–6]. Low serum Vitamin D is associated with NAFLD incidence and progression [7–9]. .The liver is also responsible for 25-hydroxylation of vitamin D, an important step in biotransformation to its active form [6].

Vitamin D functions are mediated through its receptor (VDR), which belongs to a diverse group of steroid receptor superfamily [10]. It has been shown that single nucleotide mutations within the VDR gene on the 12cen-q12 chromosome may affect the stability, quantity, and activity of the produced protein, as well as the transcription rate of the gene [11]. ApaI (RS7975232), BsmI (RS1544410), TaqI (RS731236), and FokI (RS2228570) are four common single nucleotide polymorphisms in the VDR gene [12]. The FokI variants give rise to distinct initiation sites during VDR translation and produce different VDR proteins [13].

Correction of vitamin D status in some studies has ameliorated liver steatosis and inflammatory biomarkers, but this was not true for other populations which indicates inter-individual variations in response to vitamin D treatment. This heterogeneity in response to vitamin D therapy may be attributed to VDR gene variations [14]. .There has been no study evaluating the association between the VDR FokI variants and the development of NAFLD and its response to calcitriol administration. This clinical trial research was designed to compare the effects of calcitriol intake on laboratory, inflammatory, and metabolic parameters in NAFLD patients with different variants of VDR FokI polymorphism.

## Methods

### Study population

This clinical trial was conducted on 128 fatty liver patients recruited by a gastroenterologist and liver specialist from patients referred to the Ahvaz Golestan Hospital, Iran, in 2017–2018. All recruited patients met the defined inclusion criteria for participation in the study and filled and signed a personalized form and informed consent form issued by the Committee of Ethics at Ahvaz Jundishapur University. The procedure was approved by the Committee with the code IRCT2017053034222N1 and was under the 1964 Helsinki declaration and its later amendments. The trial was also registered at the Iranian Registry of Clinical Trials (irct.ir) with the same code.

The inclusion and exclusion criteria.

Individuals between the age of 18–60 with the ultrasonography and lab data proved NAFL disease have entered the study. The inclusion criteria included elevated levels of serum alanine aminotransferase (ALT), negative diagnostic tests for hepatitis B and C, no history of alcohol intake and the exclusion criteria were no record of hepatotoxic drug and vitamin D supplements consumption in the last three months, no pregnancy and lactation conditions, and no background of malabsorption syndromes.

Individuals with a history of viral hepatitis, alpha-1 antitrypsin deficiency, autoimmune liver diseases, Wilson disease, multiple cancers, and a history of kidney stones, cardiovascular, renal, serum calcium higher than 10.6 mg/dl, and endocrine diseases were excluded from the study.

### Study design and intervention

We designed a randomized, double-blind, placebo-controlled trial to investigate the differential effect of calcitriol versus placebo in NAFLD patients with different FokI VDR variants. Baseline assessment was performed for all registered patients including anthropometric and medical examination before randomization. Patients were assigned to two groups using a randomized block design, with a block size of 6 in a 1:1 ratio using random numbers provided by a computer. The sample selection was assigned via hiding method and the single codes were used for each person. The patients and the medical staff were unaware of the types of treatment assigned to the subjects. Patients received either 0.25 μg Calcitriol (Zahrawi Co, Iran) daily or placebo for 17 weeks. The appearance and package of the placebo were the same as the drug. Patients were followed-up at 8 weeks intervals to check for their compliance with the intervention. The primary outcome was the change in liver function tests including ALT/AST activity and fatty liver indices including HSI and APRI factors. Secondary outcomes of glycemic indices and lipid factors were also measured and compared in the groups.

### Measurements

Anthropometric indices such as height and weight were measured using a Seca scale with height (Germany). Enzymatic biochemical assays were applied for glucose, lipid profile, and liver enzymes measurement. Bilirubin was measured by a photometric method and albumin was measured based on bromocresol green binding using commercial assay kits (Pars-Azmoon Co, Tehran, Iran) on the Mindray BS-200E biochemical analyzer. The Accubind Insulin ELISA kit (Monobind Co, CA, USA) was used for fasting insulin measurement. Blood platelet count was quantified using the Mindray cell counter. Homeostasis model assessment–insulin resistance (HOMA-IR) levels were calculated with the following formula: [fasting blood sugar (mg/dl) × fasting insulin (μIU/mL)] ÷ 405 [15]. Noninvasive fatty liver indices including HSI and APRI factors were determined according to the following formulas [16]:

$$ \mathrm{APRI}=\mathrm{AST}\left(\mathrm{IU}/\mathrm{L}\right)/\mathrm{AST}\ \mathrm{upper}\ \mathrm{limit}\ \mathrm{of}\ \mathrm{normal}\ \left(\mathrm{IU}/\mathrm{L}\right)/\mathrm{platelet}\ \mathrm{count}\ \left(\times 1{0}^9/\mathrm{L}\right)\times 100 $$

$$ \mathrm{HSI}=8\times \left(\mathrm{ALT}/\mathrm{AST}\ \mathrm{ratio}\right)+\mathrm{BMI}\left(+2,\mathrm{if}\ \mathrm{female};+2,\mathrm{if}\ \mathrm{diabetes}\ \mathrm{mellitus}\right). $$

### Genotyping of the VDR FokI variants

DNA extraction from the blood samples was performed using the salting-out method [17]. The ARMS (amplification refractory mutation system)- PCR method was employed to determine the different genotypes of VDR FokI polymorphism based on the method described by Lombard et al. and Soborg et al. [18, 19]. The primers were designed based on a previous study [20].

Polymerase chain reactions were performed using 7.5 μl of master mix (Amplicon, Denmark), 5 picomol of each primer (wild FF, mutant ff, common), 1 picomol of each control primer (forward and reverse), and 100 ng of template DNA in a total volume of 15 μL with a peqSTAR thermal cycler. The amplified DNA was analyzed by electrophoresis on a 3% agarose gel and was stained with the YTA DNA safe stain (YTA, Tehran, Iran). Bands were visualized by a gel documentation system (Labtech International Ltd., England).

### Statistical analysis

Statistical works were carried out with the SPSS v.22.0 (IBM Corp., NY, USA). The obtained data were reported as mean ± SD or median (IQR). A *p*-value < 0.05 was considered statistically significant. The normality of data was evaluated using the Shapiro-Wilk test. At baseline, between groups comparisons were performed using the Mann-Whitney or independent sample t-tests. A 2 × 2 ANOVA test was performed to compare the data in intervention groups before and after treatments. The genotype frequencies in the placebo and calcitriol intervention groups were compared by the Chi-square test. The interaction of the FokI genetic variants to the calcitriol effects in the placebo and calcitriol groups was analyzed with the two-way analysis of variance test.

The sample size was calculated based on the mean difference of 4.7 for ALT and according to SD values reported in previous research [21]. Considering a power of 80% and type I error of α = 0.05, 61 subjects per group should be recruited to detect this difference. A dropout rate of 10% was anticipated, so a total number of 134 were recruited to the clinical trial.

## Results

### Demographic and biochemical characteristics of the subjects

#### Baseline characteristics

**Fig. 1:**
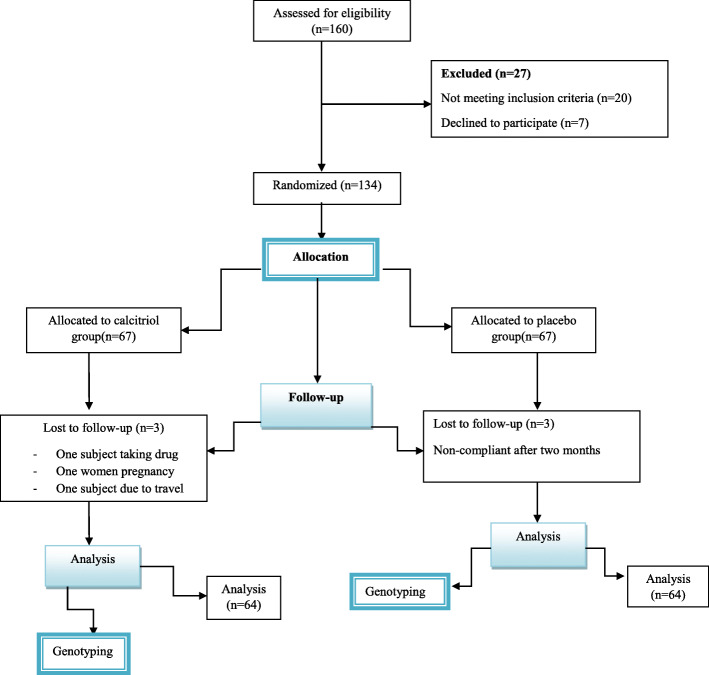
Flow diagram of the trial indicating screening, enrollment, random assignment, and follow-up of study participants

During 15 months recruitment period, 128 fatty liver subjects were registered and randomly allocated to calcitriol or placebo groups (Fig. 1). A total number of 134 patients were screened for eligibility. In two of the calcitriol and the placebo arm, sixty-four participants finished the study, with 3 patients lost to follow-up in each arm due to travel, pregnancy, drug consumption, and discontinuation of placebo intake. The mean age in both groups was 40 years and 70% of the study population were men. There were no statistically significant differences between the two groups in baseline characteristics (Table 1). Levels of vitamin D in the patients of both groups at the baseline found to be comparable (*p* = 0.3) and were below 20 ng/ml.

**Table 1:** Baseline characteristics of the calcitriol and placebo intervention groups

variable	Placebo group(*n* = 64)	Calcitriol group(*n* = 64)	*p*-value
Body weight, kg	83.3 ± 11.7	83.6 ± 13.1	0.88
BMI, kg/m^2^	29.3 ± 3.8	29.0 ± 4.7	0.67
WC, cm	101.8 ± 10.8	100.5 ± 8.8	0.44
HC, cm	107.2 ± 7.7	106.8 ± 9.2	0.78
FBS, mg/dl	94.2 ± 23.0	98.1 ± 38.3	0.49
Insulin, μIU/ml	10.8 ± 5.5	9.6 ± 4.8	0.17
Homa-IR	2.1 ± 0.9	2.0 ± 0.8	0.43
Quicki	0.33 ± 0.03	0.34 ± 0.03	0.54
HS Index	41.4 ± 5.8	41.4 ± 5.6	0.95
APRI index (IQR)	0.30 (0.22–0.38)	0.28 (0.23–0.37)	0.16
TC, mg/dl	188.0 ± 60.9	187.8 ± 46.5	0.98
TG (IQR),mg/dl	168.0 (121.5–210.5)	170.0 (134.0–236.0)	0.32
HDL cholesterol, mg/dl	41.4 ± 11.2	38.3 ± 7.9	0.07
LDL cholesterol, mg/dl	97.9 ± 27.5	96.6 ± 25.9	0.77
AST,IU	29.2 ± 15.6	27.1 ± 8.7	0.35
ALT (IQR), IU	45.5 (32.8–57.0)	23.0 (33.5–51.0)	0.53
GGT, IU	35.2 ± 17.8	37.2 ± 21.7	0.57
ALK, IU	200.2 ± 52.6	197.3 ± 54.9	0.76
Vitamin D, ng/ml	17.5 ± 7.7	19.1 ± 9.6	0.29
Hs-CRP (IQR), μg/ml	2.9 (1.1–5.5)	2.2 (1.1–4.8)	0.99
Leptin (IQR), ng/ml	11.6 (6.6–19.7)	9.9 (5.5–22.5)	0.30
Adiponectin (IQR), μg/ml	18.6 (13.6–28.2)	18.0 (10.8–24.4)	0.89

Values are expressed as the mean ± SD or median (IQR). *IQR* interquartile range, *BMI* Body mass index, *WC* Waist circumference, *HC* Hip circumference, *FBS* Fasting blood glucose, *Homa-IR* Homeostatic Model Assessment for Insulin Resistance, *HS index* Hepatic steatosis index, *APRI* AST to Platelet Ratio Index, *TC* total cholesterol, *TG* Triglyceride, *HDL-c* High-density lipoproteins- cholesterol, *LDL-c* Low density lipoprotein- cholesterol, *AST* aspartate aminotransferase, *ALT* Alanine aminotransferase, *GGT* Gamma-glutamyl transferase, *ALK* Alkaline phosphatase, *Hs-CRP* High-sensitivity C-reactive protein

#### Anthropometric measurements

After 17 weeks calcitriol treatment, there were no significant changes in anthropometric parameters compared to the baseline and in comparison to the placebo group following treatments (Table 2).

**Table 2:** Values of demographic and biochemical variables before and after calcitriol and placebo interventions

Variable	Baseline		Week 16		*P* value		
	Placebo	Calcitriol	Plasebo	Calcitriol	Group	Time	Group * Time
Weight,kg	83 ± 11.7	83 ± 13.1	82 ± 12.5	83 ± 12.8	**0.840**	**0.746**	**0.781**
BMI, kg/m^2^	29.1 ± 3.9	29.1 ± 4.7	28.6 ± 4.2	28.8 ± 4.5	**0.595**	**0.754**	**0.812**
WC, cm	101 ± 10.9	100 ± 8.9	100 ± 11.9	100 ± 9.1	**0.503**	**0.854**	**0.714**
HC, cm	107 ± 7.6	106 ± 9.2	106 ± 8.6	106 ± 9.1	**0.860**	**0.512**	**0.872**
TC, mg/dl	188 ± 62.0	187 ± 46.5	187 ± 41.0	179 ± 41.8	**0.441**	**0.827**	**0.783**
TG.mg/dl	180 ± 80.9	194 ± 100.1	178 ± 92.6	177 ± 73.4	**0.502**	**0.814**	**0.501**
HDL-c, mg/dl	40.9 ± 9.3	38 ± 7.9	40.8 ± 11.9	36 ± 9.8	**0.001**	**0.262**	**0.990**
LDL-c, mg/dl	96 ± 25.9	94 ± 23.4	103 ± 22.3	99 ± 27.4	**0.551**	**0.173**	**0.806**
FBS, mg/dl	94 ± 24.0	98 ± 38.4	94 ± 19.9	97 ± 20.0	**0.881**	**0.908**	**0.813**
Insulin, μIU/ml	10.6 ± 5.6	9.2 ± 4.8	10.7 ± 5.7	9.3 ± 4.2	**0.737**	**0.646**	**0.761**
Homa-IR	2.1 ± 0.9	2.0 ± 0.9	2.0 ± 0.8	2.0 ± 0.9	**0.546**	**0.449**	**0.879**
Quicki	0.3 **±** 0.03	0.3 ± 0.03	0.3 ± 0.03	0.3 ± 0.03	**0.433**	**0.343**	**0.672**
AST, IU	27 ± 12.6	27 ± 8.7	27 ± 14.5	23 ± 6.1	**0.95**	**0.129**	**0.713**
ALT, IU	47 ± 28.5	43 ± 16.1	35 ± 23.8	30 ± 11.4	**0.209**	**0.000**	**0.650**
GGT, IU	33.5 ± 12.5	35.8 ± 21.3	30.6 ± 21.1	35.9 ± 34	**0.245**	**0.767**	**0.190**
ALK, IU	198 ± 50.1	197 ± 54.9	188 ± 50.7	169 ± 47.3	**0.023**	**0.004**	**0.030**
HSI Index	41.5 ± 5.8	41.3 ± 5.7	38 ± 6.1	38.2 ± 6.0	**0.390**	**0.006**	**0.647**
APRI index	0.3 ± 0.1	0.3 ± 0.1	0.3 ± 0.0	0.2 ± 0.0	**0.154**	**0.242**	**0.983**
Hs-CRP, μg/ml	3.7 ± 3.5	4.5 ± 6.0	3.4 ± 4.2	3.9 ± 5.2	**0.320**	**0.495**	**0.907**
Leptin, ng/ml	17.5 ± 20	14.6 ± 13.9	11.3 ± 9.7	10.6 ± 12.1	**0.798**	**0.001**	**0.655**
Adiponectin, μg/ml	20.5 ± 11.3	21 ± 14.6	18.7 ± 9.7	17.5 ± 10.0	**0.57**	**0.006**	**0.282**

Values are expressed as the mean ± SD. *BMI* Body mass index, *WC* Waist circumference, *HC* Hip circumference, *FBS* Fasting blood glucose, *Homa-IR* Homeostatic Model Assessment for Insulin Resistance, *HS index* Hepatic steatosis index, *APRI* AST to Platelet Ratio Index, *TC* total cholesterol, *TG* Triglyceride, *HDL-c* High-density lipoproteins- cholesterol, *LDL-c* Low density lipoprotein- cholesterol, *AST* aspartate aminotransferase, *ALT* Alanine aminotransferase, *GGT* Gamma-glutamyl transferase, *ALK* Alkaline phosphatase, *Hs-CRP* High-sensitivity C-reactive protein

#### Lipid profile and glycemic indices

As shown in Table 2, lipid profile changes were not significant in response to the calcitriol treatment. In comparison to the placebo, TG decreased 17.1 mg/dl more in response to calcitriol; however, none of the lipid variables showed a significant change.

Within group and between group analyses of glycemic parameters changes also showed no significant alterations following treatments.

#### Liver parameters and indices

In the calcitriol group, AST, ALT, and ALP presented 3.7, 13.3, and 27.5 IU reduction respectively compared to the baseline (*p* < 0.001 for all). But in comparison to the placebo group, only ALP response to calcitriol remained significant (17.5 IU. *P* = 0.02) (Table 2).

The fatty liver index HIS decreased 2.5 and 3.0 units in the placebo and calcitriol groups, respectively, at the end of treatments (*p* < 0.01), but the changes were not statistically different between groups (*p* = 0.35). Additionally, the APRI index was only attenuated in the calcitriol group vs. baseline (0.3 unit, *p* < 0.001). But the change of APRI against placebo was not significant.

#### Inflammatory variables and adipokines

Hs-CRP, leptin, and adiponectin were measured in the NAFL patients with regard to liver inflammation. None of these variables showed a significant change in both arms at the end of the study.

### Distribution of VDR 10735810 C > T (FokI) gene polymorphism

The allele and genotype frequencies of VDR gene FokI are shown in Table 3. Chai square analysis confirmed that the genotypes were similarly distributed in the placebo and calcitriol groups. In the total population of the study, the FF genotype showed the highest frequency (53.1%) followed by the Ff genotype (45.3%). The minor genotype with 1.6% frequency was the ff homozygous, which was excluded from the genotype-calcitriol effects interaction analysis due to the limited number of subjects with this genotype.

**Table 3:** The allele and genotype frequencies of VDR FokI gene polymorphism

Group	Genotypes			Allele frequencies		***P***-value	***χ***^**2**^
	FF	Ff	ff	F	f		
**Placebo**	31 (48.4%)	32 (50%)	1 (1.6%)	73%	27%		
**Calcitriol**	37 (57.8%)	26 (40.6)	1 (1.6%)	78%	22%	0.563	1.150

The interaction of the FokI FF and Ff genotypes to the calcitriol effects was tested with the Two-way ANOVA. As presented in Table 4, the two-way ANOVA demonstrated a significant interaction of this polymorphism with the ALP response to the calcitriol treatment. Specifically, the decrease in the ALP activity was higher in the subjects of the calcitriol group with the Ff genotype (*p* = 0.05).

**Table 4:** Differential changes of variables following placebo and calcitriol treatments in subjects with FF and Ff genotypes of the VDR FOKI polymorphism

Variable	FOKI FF genotype(***n*** = 68)		FOKI Ff genotype (***n*** = 58)		***p***-value		
	placebo	Calcitriol	placebo	Calcitriol	Group	Genotype	Interaction
**weight, kg**	−0.16 ± 2.4	0.24 ± 1.8-	− 1.0 ± 2.3	0.5 ± 3.8-	0.67	0.24	0.57
**BMI, kg/m**^**2**^	0.00 ± 0.75-	−0.21 ± 1.0	− 0.36 ± 0.8	0.2 ± 1.3-	0.89	0.35	0.34
**WC, cm**	−0.8 ± 1.8	0.12 ± 2.8	−0.8 ± 1.9	−0.3 ± 2.0	0.10	0.58	0.55
**HC, cm**	0.16 ± 3.4	0.8 ± 3.6-	−0.6 ± 1.4	− 0.6 ± 3.0	0.37	0.58	0.39
**FBS, mg/dl**	−2.0 ± 13.5	2.0 ± 19.3	1.3 ± 9.1	−4.6 ± 42.8	0.82	0.71	0.27
**Insulin**	−0.05 ± 6.8	0.12 ± 6.4	0.2 ± 6.7	0.1 ± 4.6	0.97	0.90	0.91
**Homa-IR**	−0.2 ± 0.7	0.1 ± 1.3	0.08 ± 0.9	− 0.2 ± 1.0	0.74	0.90	0.09
**TC, mg/dl**	−4.5 ± 60.1	−7.8 ± 33.7	1.6 ± 46.1	−8.8 ± 47.4	0.43	0.86	0.68
**TG.mg/dl**	−8.1 ± 95.1	−29.2 ± 99.8	5.3 ± 83.2	1.0 ± 82.0	0.46	0.20	0.62
**HDL -C, mg/dl**	−2.2 ± 10.9	−3.1 ± 9.7	1.4 ± 8.8	−1.2 ± 11.0	0.34	0.14	0.66
**LDL-C, mg/dl**	7.5 ± 26.1	5.0 ± 21.3	7.4 ± 23.7	6.4 ± 27.4	0.71	0.89	0.87
**AS, IU**	0.08 ± 15.3	−4.9 ± 7.3	−1.2 ± 8.5	−2.4 ± 8.4	0.10	0.74	0.31
**ALT, IU**	−12.6 ± 22.3	− 15.3 ± 19.9	−10.2 ± 17.4	−11.2 ± 12.2	0.58	0.34	0.80
**GGT, IU**	0.18 ± 28.9	−5.3 ± 13.5	−5.6 ± 15.6	7.6 ± 48.5	0.50	0.51	0.09
**ALK, IU**	−26.3 ± 74.7	−23.0 ± 38.1	2.2 ± 54.5	−33.3 ± 42.8	0.11	0.36	0.05
**Vitamin D**	−0.1 ± 6.4	2.1 ± 9.1	1.1 ± 6.4	0.73 ± 5.9	0.13	0.39	0.86
**Hs-CRP,μg/ml**	0.12 ± 3.2	0.0 ± 2.9	−0.38 ± 4.7	−1.3 ± 6.0	0.53	0.28	0.63
**Leptin, ng/ml**	−6.3 ± 17.9	−2.6 ± 12.6	−6.6 ± 21.1	−6.2 ± 13.3	0.53	0.55	0.61
**Adiponectin, μg/m**	−4.5 ± 12.2	−4.4 ± 14.2	0.05 ± 12.8	−1.8 ± 7.8	0.75	0.13	0.69
**HSI**	−2.2 ± 3.8	3.1 ± 4.5-	− 2.7 ± 4.9	−2.7 ± 4.1	0.56	0.92	0.54
**APRI**	0.0 ± 0.2	−0.04 ± 0.1	0.0 ± 0.1	−0.03 ± 0.1	0.20	0.95	0.73

Values are expressed as the mean ± SD. *BMI* Body mass index, *WC* Waist circumference, *HC* Hip circumference, *FBS* Fasting blood glucose, *Homa-IR* Homeostatic Model Assessment for Insulin Resistance, *HS index* Hepatic steatosis index, *APRI* AST to Platelet Ratio Index, *TC* total cholesterol, *TG* Triglyceride, *HDL-c* High-density lipoproteins- cholesterol, *LDL-c* Low density lipoprotein- cholesterol, *AST* aspartate aminotransferase, *ALT* Alanine aminotransferase, *GGT* Gamma-glutamyl transferase, *ALK* Alkaline phosphatase, *Hs-CRP* High-sensitivity C-reactive protein

## Discussion

To our knowledge, this is the first randomized controlled trial (RCT) designed to evaluate the calcitriol effects on NAFLD patients with regard to different genotypes of vitamin D receptor FokI polymorphism. In our study the baseline level of vitamin D in all the enrolled patients was deficient, suggesting that it might be involved in the development of the disease. Epidemiological studies have also shown that NAFLD is associated with low vitamin D levels in serum, and vitamin D deficiency is 26% more common in NAFLD patients than healthy subjects [8, 22]. As NAFLD is a common hepatic presentation of metabolic syndrome, Schuch et al. have investigated and reported a relationship between vitamin D receptor gene polymorphisms and the component of metabolic syndrome [23]. However, a causal relationship between them has been argued due to the cross-sectional nature of these studies. Besides, recent findings of a meta-analysis involving 974 NAFLD patients revealed that histologic severity in these patients was not associated with vitamin D levels [24]. Another study on 9182 Chinese participants showed no causal association between vitamin D and NAFLD using bi-directional Mendelian randomization (MR) analysis [25].

Our results indicated that when the calcitriol effects were compared to the placebo group, only ALP decrease was found significant. In the present study, all the patients were initially given weight loss and diet intervention recommending restriction of high-carbohydrate, high-fat foods, and an increase in physical activity. The partial amelioration of anthropometric and fatty liver parameters in the placebo and calcitriol groups compare to the baseline can be attributed to these lifestyle modifications.

The primary risk factor in the development of NAFLD is insulin resistance that is involved in the formation of hepatic steatosis, oxidative stress, and lipotoxicity [26–28]. Contrary to some reports of improvement in glycemic and inflammatory parameters in response to vitamin D treatment [3, 4], in our study calcitriol administration did not alleviate glycemic or metabolic status. This result is in accordance with a preceding meta-analysis of 35 RCTs including 43,407 patients that concluded vitamin D could not attenuate insulin resistance in diabetic patients and did not prevent diabetes in non-diabetic subjects [29]. Altogether, based on the present data a conclusion of glycemic status improvement by raising vitamin D concentrations cannot be made. There are further investigations that confirm the effect of vitamin D supplementation on improving lipid profile and inflammatory mediators in comparison with placebo [3].

Notwithstanding these reports, the results of our study and more recent controlled trials did not support the argument that vitamin D can improve lipid profile and NAFLD associated hepatic inflammation [30].

Likewise, the decrease in leptin level compared to the baseline in our study in both calcitriol and placebo groups could be due to weight loss and fat mass reduction because its changes were not statistically significant when compared to the placebo group and adjusted for the anthropometric changes. These results, in accordance to the recent meta-analysis studies [31], suggest that calcitriol treatment at the therapeutic doses which was applied in this study could not provide further efficacy than the basic weight loss, diet, and exercise recommendations.

According to the previous meta-analysis and systematic reviews on the efficacy of Vitamin D supplementation against NAFLD [3, 29], most RCTs were carried out using cholecalciferol for vitamin D intervention, but considering our objective to assess the differential VDR FokI genotypes responses to vitamin D in the context of NAFLD, we made our interventions using calcitriol which is the immediate ligand for VDR to produce biologic effects. The recommended initial safe dose of calcitriol treatment is 0.25 mcg daily [32]. Higher doses might put recipients at the risk of toxicity and hypercalcemia and increased dropouts as a result.

Hepatic VDR expression is up-regulated in NAFLD patients and genetic variation in this receptor can result in inter-individual differences in response to vitamin D [33]. VDR genetic variants may alter the host response to vitamin D treatment. In this regard, people with homozygous ff genotype of FokI were considered as low responders to vitamin D treatment [13]. Our results showed that the VDR FokI homozygous F and heterozygous variants did not have significant interactions with the calcitriol effects except for ALP that displayed significant changes between groups and genotypes in which the Ff genotype was found more responsive to the calcitriol treatment. This exclusive ALP response to vitamin D has been reported in earlier studies. A meta-analysis study conducted on 9 trials concluded that vitamin D intake had no efficacy on NAFLD treatment except for ALP which was significantly reduced [34]. Increased ALP level in serum is a marker of vitamin D insufficiency. However, significant correlations between serum 25-hydroxyvitamin D and ALP levels were not generally established [35]. But when taking VDR FokI variants into account, their correlation was found significant in the FF-type, but not in the ff-type [36]. It seems that the primary targets of vitamin D specifically may respond variably to calcitriol with regard to VDR FokI variants.

To our knowledge, this is the first study addressing the VDR FokI genotypes interactions in the outcome of vitamin D intervention. However, the following limitations should be acknowledged regarding the current study. The methods that were used for NAFLD diagnosis could not distinguish between simple steatosis from NASH and no judgment can be made regarding changes in liver inflammation, cellular injury, and fibrosis compared to the liver biopsy as a gold standard. We also could not take up high doses of calcitriol for intervention due to the adverse effects, increased risk of toxicity, and dropouts. Another limitation was the sample size which did not provide sufficient subjects with the ff genotype due to the low frequency of this variant in our population as were observed by Montazeri-Najafabady et al. [37]. Therefore, based on low frequency of the ff genotype in the general population, we could not conclude that the ff genotype may be a protective factor against the development of NAFLD, and also further studies with higher sample sizes are required to judge the implication of this and other genetic variants of VDR in the outcomes of vitamin D therapy in NAFLD.

## Conclusion

Although vitamin D deficiency is known as an independent risk factor for NAFLD, when the disease is fully developed, the afterward vitamin D supplementation cannot correct the negative consequences of its chronic deficiency in fatty liver. The FF and Ff variants of VDR FokI polymorphism did not interact with the effects of calcitriol on fatty liver, but the ALP response, as a direct associated parameter to calcitriol treatment was connected to the FokI polymorphism, with the Ff being the more responsive genetic variant.

## Data Availability

The datasets used and/or analysed during the current study are available from the corresponding author on reasonable request.

## Abbreviations

NAFLD: Non-alcoholic fatty liver disease
NASH: Nonalcoholic steatohepatitis
HCC: Hepatocellular carcinoma
VDR: Vitamin D receptor
ALT: Alanine aminotransferase
AST: Aspartate aminotransferase
ALP: Alkaline phosphatase
HSI: Hepatic steatosis index
APRI: Aspartate aminotransferase to platelet ratio index
HOMA-IR: Homeostasis model assessment–insulin resistance
ARMS: Amplification refractory mutation system
BMI: Body mass index
ANOVA: Two-way analysis of variance
Hs-CRP: High-sensitivity C-reactive protein
MR: Mendelian randomization
